# Microwave-assisted graphene oxide/carbon spheres with silver nanoparticles: dual catalyst for peroxide detection and antibacterial use

**DOI:** 10.1098/rsos.242263

**Published:** 2025-08-13

**Authors:** Thanh Long Phan, Thanh Tho Le, Van-Dat Doan, Huynh Anh Vu Truong, Van Tan Le

**Affiliations:** ^1^ Faculty of Chemical Engineering, Industrial University of Ho Chi Minh City, Ho Chi Minh City, Vietnam; ^2^ Center of Analytical Services and Experimentation Ho Chi Minh City, Ho Chi Minh City, Vietnam

**Keywords:** silver nanoparticles, carbon spheres, graphene oxide, peroxidase-like activity, antibacterial activity

## Abstract

In this study, a novel nanocomposite was rationally engineered by embedding silver nanoparticles (AgNPs) into tannic acid-derived carbon spheres (Cs), followed by their distribution on graphene oxide (GO) sheets (AgNPs@Cs-TA@GO), aiming to enhance catalytic and antibacterial performance. The fabrication process was expedited using a microwave-assisted technique. The AgNPs@Cs-TA@GO nanocomposite with the core based on AgNPs with an average diameter of 21 nm showed significant peroxidase-like activity by oxidizing 3,3′,5,5′-tetramethylbenzidine to its oxidized form in the presence of H_2_O_2_. This compassionate H_2_O_2_ colorimetric detection allowed for quick identification of H_2_O_2_ in a broad range (0.6–9.0 ppm) with a low detection limit of 0.2 ppm (*S*/*N* = 3). The viability of this method was confirmed by qualitatively detecting H_2_O_2_ residual in aquaculture water samples. Additionally, the antibacterial assays, conducted using the agar well diffusion method demonstrated pronounced inhibitory effects against *Vibrio parahaemolyticus* ATCC 17802 (Gram-negative) and *Staphylococcus aureus* ATCC 25923 (Gram-positive), with inhibition zones of 46.1 and 49.3 mm, respectively. The rapid microwave-assisted incorporation of AgNPs into Cs within the GO network not only improves synthesis duration but also the catalytic and antibacterial performances as a result of minimized coagulation and promoting electron transfer on the AgNPs’ surface.

## 1. Introduction

Silver nanoparticles (AgNPs) are commonly used in the manufacture of many types of composites for various applications in different fields, including biomedical and environmental industries, owing to their strong antibacterial and catalytic capabilities [1–4]. Incorporating AgNPs onto a matrix can improve the structural properties of the nanoparticles, leading to stable composites with AgNPs of small diameters, large surface areas and strong durability [5]. AgNPs could be attached to a polymer network to reduce clumping and enhance the outstanding antibacterial and catalytic properties of AgNPs [6]. These Ag@polymer composites have also shown promising optical sensitivity to heavy metal ions, catalytic activity in organic pollutant removal and multifunctional antibacterial behaviour, highlighting their potential in diverse applications [7,8].

A key aspect of catalytic applications is peroxidase-like activity, typically evaluated through the oxidation of 3,3′,5,5′-tetramethylbenzidine (TMB). This reaction serves as a widely adopted model in biosensing platforms employing peroxidase mimetics, such as nanomaterials and G-quadruplex-based DNAzymes for the detection of metal ions, nucleic acids, proteins and cancer cells [9]. Beyond biosensing, TMB oxidation is also used to evaluate a broad range of biological and inorganic catalysts, including haemoglobin, ferritin, iron, nitrite and synthetic peroxidase analogues [10]. It has additionally been applied in identifying oxidizing agents (e.g. H_2_O_2_, chlorine, bromate) and reducing agents (e.g. glutathione, Au(I), Mn²^+^ and graphene-based radicals) [11]. Furthermore, TMB oxidation enables the indirect quantification of analytes such as glucose, melamine and organophosphates by monitoring H_₂_O_₂_ generated in oxidase-catalysed reactions [12,13]. Given the extensive use of hydrogen peroxide in food, medical and industrial applications [14], its precise detection is essential. To this end, various analytical techniques have been developed, including colorimetric, titrimetric, fluorescence-based and electrochemical methods [15,16], among which colorimetric assays are especially favoured for their simplicity, rapidity and cost-efficiency [17,18].

Graphene oxide (GO) has recently emerged as a promising platform for enhancing the performance of AgNPs. GO provides a large surface area and excellent conductivity, making it an ideal support for dispersing AgNPs and enhancing their functionality [19]. Previous investigations, exemplified by the work conducted by Nguyen *et al. *[20], have showcased the fabrication of silver-reduced graphene oxide (Ag@rGO) composite aimed at sustained antibacterial applications, with demonstrated effectiveness of up to 99.99% for *Staphylococcus aureus*, *Escherichia coli* and *Pseudomonas aeruginosa*. Ag@rGO nanomaterials also revealed effective catalytic activity based on the electron transfer mechanism with more than 99% of dyes reduced in their aqueous solution after 30 min. In 2020, Menazea & Ahmed confirmed that the decoration of GO by silver and nanoparticles is growing, owing to their use in science and technology. GO sheets with surfaces highly anchored by sphere-shaped AgNPs have enormous potential for antibacterial application in a wide variety of biomedical uses [21]. Furthermore, Zhang *et al*. explored the catalytic application of a nanocomposite based on AgNPs incorporated with reduced GO and confirmed that the composite exhibited good anti-interference, long-term stability and repeatability for H_2_O_2_ sensing [22]. In 2022, Joshi *et al*. used the carbonization method to dope AgNPs in carbon spheres (Cs) and confirmed that the nanocomposite can be considered as a potential biocidal material, especially for *Escherichia coli* and *Bacillus subtilis* [23]. In 2017, Wang *et al*. successfully fabricated a graphene-colloidal carbon sphere composite decorated with AgNPs via a hydrothermal method and demonstrated its effectiveness in developing a highly selective and sensitive electrochemical sensor for hydrogen peroxide detection with a fast response time and a low detection limit [24].

Alongside catalytic detection, AgNPs and their composites have also proved effective in combating pathogenic bacteria. *Vibrio parahaemolyticus (V. para*), a marine bacterium prevalent in aquaculture, has been a major cause of foodborne illness and shrimp disease (AHPND) in Southeast Asia, including Vietnam, since the 1950s [25,26]. Similarly, *Staphylococcus aureus (S. aureus*), a common part of the human microbiota, is responsible for a wide spectrum of infections and is increasingly difficult to treat owing to antibiotic resistance [27–30]. The development of advanced antibacterial nanocomposites is therefore critical to addressing these biological threats.

Porous carbon sphere-based composites have attracted growing attention as nanocarriers in recent years, particularly for their potential in enhancing catalytic and antibacterial performance [31]. Their widespread appeal stems from advantageous properties such as low density, excellent electrical conductivity, large specific surface area, tunable porosity and cost-effectiveness [32]. Owing to these features, Cs and their composites have been widely applied in various fields, demonstrating marked improvements in functional performance [33,34]. Recent research efforts have increasingly focused on the nanoscale functionalization of carbon sphere-based platforms, positioning them as promising candidates for advanced catalytic and antimicrobial systems through strategic integration with metal nanoparticles [35].

Microwave-assisted synthesis has emerged as a rapid and eco-friendly technique for fabricating metal nanoparticle-based composites. This method enables uniform heating and precise control over particle morphology while reducing reaction time and solvent use [36–40]. Numerous studies have highlighted the potential of microwave-assisted synthesis in the efficient fabrication of metal nanoparticles and their nanocomposites. For instance, gold nanoparticles (AuNPs) were successfully synthesized in a one-pot microwave-assisted process using *Myristica fragrans* leaf extract. The resulting biogenic AuNPs displayed excellent crystallinity, a narrow size distribution and high catalytic efficiency in the degradation of 4-nitrophenol and methyl orange [41]. Similarly, a rapid microwave-assisted synthesis enabled the fabrication of AgNPs and AgNP-decorated/holocellulose nanofibrils within 1 min, producing a nanocomposite with superior performance in Hg(II) detection and dye reduction [42].

Despite growing research in AgNP-based materials, the microwave-assisted synthesis of a nanocomposite combining AgNPs, Cs and GO designed for both antibacterial and catalytic performance remains largely unexplored.

In this study, a novel nanocomposite was developed by embedding AgNPs into Cs via a facile, rapid microwave-assisted synthesis. Notably, tannic acid (TA) was employed for the first time as both a carbon precursor and a green reducing agent, enabling the simultaneous formation of Cs-TA and the *in situ* reduction of silver ions into uniformly distributed AgNPs (AgNPs@Cs-TA). This dual functionality of TA facilitated the strong adhesion of AgNPs onto the carbon matrix without requiring additional stabilizers or complex procedures. The resulting AgNPs@Cs-TA was subsequently anchored onto GO sheets using an ultrasonication-assisted approach, yielding the ternary nanocomposite (AgNPs@Cs-TA@GO). The strategic confinement of AgNPs within/on the porous Cs and their dispersion across the conductive GO network was designed to minimize AgNPs-loaded Cs aggregation and enhance surface electron transfer.

## 2. Material and methods

### 2.1. Materials

All the substances used in this study were of analytical quality and were used without additional purification. The chemicals used were TA (C_76_H_52_O_46_, 99.5%), graphite fine powder (99.5%), silver nitrate (AgNO_3_, 99.8%), NH_3_ solution (25%), H_2_O_2_ solution (30%) and red-TMB, obtained from Merck (Singapore) and Acros Organic Company (Belgium). Deionized water was used in all tests to ensure uniformity and precision. The bacterial strains, including *S. aureus* subsp. Aureus ATCC 25923 and *V. para* ATCC 17802 were acquired from Microbiologics company and stored in the Department of Biotechnology, Centre for Analytical Services and Experimentation in Ho Chi Minh City, Vietnam.

### 2.2. Carbon sphere fabrication

An exact amount of 1 g of TA was dissolved in 25 ml of distilled water and sonicated for 1 h to ensure uniform mixing. The resulting solution, with a TA concentration of 0.04 g ml^−1^, was then transferred to a 110 ml microwave digestion vessel equipped with temperature and pressure control. Microwave irradiation was applied at 70% power using a MARS 6 system (CEM Corporation, USA) for 10 min, yielding a free-flowing black-brown solution. The MARS 6 system offers unparalleled efficiency for nanoparticle synthesis, using advanced One Touch Technology to simplify and enhance microwave high-quality nanoparticle production. With a power output of up to 1800 W and precise control via a microcomputer, it can deliver rapid, uniform heating of polar liquids or ionic solutions, enabling faster reaction times. After synthesis, the obtained suspension was then evaporated at room temperature and washed several times with distilled water for future use.

### 2.3. Incorporation of silver nanoparticals into carbon spheres

The manufacture of AgNPs@Cs-TA was achieved using a microwave-assisted approach with TA as the primary ingredient. The experiment involved using Cs-TA and AgNO_3_, while focusing on optimizing three main factors: the ratio of AgNO_3_ to TA (1 : 2, 1 : 5, 1 : 10, 1 : 20), synthesis length (0, 2, 5, 10 and 15 min) and microwave power (0, 20, 50, 70 and 100%). AgNPs immobilized on Cs, known as AgNPs@Cs-TA, were produced via a one-step microwave-redox reaction in a microreactor. The synthesis procedure included dispersing 0.1 g of AgNO_3_ in 10 ml of 0.1 M NH_3_ aqueous solution. Subsequently, 10 ml of this solution was introduced into microreactors with the carbon sphere solution fabricated from 0.2, 0.5, 1.0 and 2.0 g of TA. The microreactors were then exposed to microwave irradiation to produce AgNPs@Cs using optimal parameters, such as the ratio of AgNO_3_ to TA, microwave power and synthesis duration. The synthesis process notably excluded the external use of any surfactants, reducing agents or organic solvents. After the reaction was finished and cooled down naturally, the aqueous solution of AgNPs@Cs-TA was washed with deionized water and ethanol. It was then air-dried for further analysis and use.

### 2.4. Synthesis of graphene oxide

GO was produced by employing a revised Hummers’ method described by Hummers & Offeman in 1958 [43]. One gram of graphite powder was combined with 150 ml of 98% sulfuric acid and 1.0 g of sodium nitrate in a 1.0 l flask on a magnetic stirrer. The mixture underwent mechanical stirring for 24 h at 60°C, then was cooled down to room temperature. Later, 5.0 g of KMnO_4_ was added to the solution, and stirring was continued for another 2 h. The mixture was diluted with deionized water to a total volume of 1.0 l, while keeping the solution in an ice bath. For over 30 min, 10 ml of 30% hydrogen peroxide solution was added dropwise while stirring until the mixture changed to a bright yellow hue. The solution was filtered and then dispersed using an ultrasonic sonifier for 15 min in a solution containing 100 ml of 10 wt% HCl to eliminate metal ions and residual acid. The resulting mixture was dispersed in 2.0 l of deionized water to form a GO aqueous suspension. The GO product was obtained by centrifuging the suspension at 8500 r.p.m. for 30 min. The solid GO was subsequently dried in an oven at 60°C overnight.

### 2.5. Anchoring AgNPs@Cs-TA onto graphene oxide sheets

The AgNPs@Cs-TA@GO composite was synthesized by combining AgNPs@Cs-TA and GO using ultrasonic irradiation for 30 min. Various ratios of GO to AgNPs@Cs-TA (5, 10, 20 and 30%) were tested to optimize the AgNPs@Cs-TA@GO material. The optimization technique included putting different quantities of GO in beakers with specified concentrations of AgNPs coated with cesium–tantalum. Successively, 0.5, 0.1, 0.2 and 0.3 g of GO were added to beakers with 0.95, 0.90, 0.8 and 0.7 g of AgNPs@Cs-TA, respectively. Afterwards, 50 ml of distilled water was added to each beaker and ultrasonic agitation was carried out at 30°C for 30 min. After synthesis, the resulting composite was repeatedly washed with deionized water and ethanol and then centrifuged to eliminate any residual, unbound GO or AgNP@Cs-TA.

### 2.6. Characterizations

Examination of the synthesized AgNPs@Cs-TA@GO composite was done by using several analytical techniques to gain distinct insights into the material’s structure and behaviour. Fourier transform infrared (FTIR) spectroscopy was performed using a Nicolet iS50 instrument (Thermo, USA) to analyse the solid samples over a spectral range of 4000–400 cm^−1^. FTIR analysis assisted in recognizing the organic functional groups, providing important details about chemical bonding and composition. X-ray diffraction (XRD) examination was conducted with the Shimadzu 6100 X-ray diffractometer from Japan. The instrument functioned at 40 kV and 30 mA, using CuK_α_ radiation with a wavelength of 1.5406 nm. The scanning settings consisted of a scanning speed of 0.05° s^−1^ and a step size of 0.02°, spanning the 2θ range from 5° to 80°. The XRD set-up allowed for the analysis of the crystalline structure and phase composition of the samples, offering vital information about their atomic arrangements and crystallographic features. The Debye–Scherrer equation was used to determine the average crystallite sizes of AgNPs@Cs-TA@GO. The equation establishes a relationship between the crystallite size (*D*), XRD peak width (*β*), X-ray wavelength (*λ*) and diffraction angle:


(2.1) $$A=\frac{K\lambda }{\beta cos\theta }.$$

The AgNPs@Cs-TA@GO composite was analysed for its morphological characteristics using high-resolution transmission electron microscopy (HR-TEM, JEOL JEM-2100, Japan), scanning electron microscopy equipped with energy-dispersive X-ray spectroscopy (SEM-EDX, Hitachi 4800). Thermogravimetric analysis (TGA) was used to study the thermal stability and breakdown characteristics of the materials. The samples’ weight loss was on a TGA55 instrument from TA Instruments, USA. The samples were heated from 50 to 900°C at a rate of 10°C min^−1^ under a 40 ml min^−1^ nitrogen purge. Dynamic light scattering (DLS) analysis was conducted on a colloidal solution using a nanoPartica Horiba SZ-100 instrument (Japan). This method allowed for the calculation of the zeta potential and the particle size distribution, offering vital insights into the surface charge and stability of the colloidal system.

### 2.7. Antibacterial assays

The composite’s antibacterial effectiveness was tested against *V. para* ATCC 17802 (Gram-negative) and *S. aureus* ATCC 25923 (Gram-positive). The bacterial strains were stored in cryo-bank tubes at −70°C, provided by the Microbiology Department at the Centre of Analytical Service and Experimentation in Ho Chi Minh City, Vietnam. The bacterial strains were reactivated by culturing them on nutrient agar (Merck, 105450) at 37°C for 24 h before experimentation. Antibacterial tests were performed on Cs-TA, AgNO_3_@Cs-TA, AgNPs@Cs-TA and AgNPs@Cs-TA@GO using the agar well diffusion method based on the Kirby–Bauer test. Bacteria were grown in brain heart infusion broth (Merck, 110493) until the cultures reached a turbidity of 0.5 McFarland Standard. Next, 0.1 ml of bacterial culture was placed on the surface of Muller Hinton agar plates from Merck (103 872). Aseptically punched holes with a 6 mm diameter were created, and the antimicrobial agent at the desired concentration was added to each well. A volume of 200 μl water and 25 ppm chlorine dioxide (ClO_2_) was used as negative and positive controls, respectively, for comparison. Two hundred microlitres of Cs-TA (20%), AgNO_3_@Cs-TA (1%), AgNPs@Cs-TA (50 ppm) and AgNPs@Cs-TA@GO (at concentrations of 50, 25, 12.5, 6.25 and 4.16 ppm) were also included in the wells. After incubation at 37°C for 24 h, the diameters of the bacterial inhibition zones were measured using the InGenius3 Bio Imaging System (Syngene, UK), which is equipped with a 5 megapixel camera, overhead white LED illumination and GeneTools analysis software. The diameters of the inhibition zones against bacteria for the materials were reassessed at 8, 24 and 48 h.

### 2.8. Peroxidase-like activity and hydrogen peroxide assay

The catalytic activity of AgNPs@Cs-TA@GO composite as a peroxidase mimic was evaluated by catalysing the oxidation of TMB using H_2_O_2_. The experimental procedure included combining 0.5 mg of AgNPs@Cs-TA@GO with 1.0 ml of H_2_O_2_ (600 ppm) and 2.0 ml of TMB (1.0 mM) in a quartz cuvette at pH 7. A UV–Vis Shimazdu 1900i spectrophotometer was used to monitor the reaction after 5 min. The peroxidase-like activity of AgNPs@Cs-TA@GO and AgNPs@Cs-TA composites was studied to determine the optimal reaction conditions by varying parameters such as pH (2.0–13), reaction time (0–60 min) and material weight (0.1–1.0 mg). Various systems were tested for their peroxidase mimetic performance using different combinations such as H_2_O_2_ + TMB, TMB + AgNPs@Cs-TA, TMB + AgNPs@Cs-TA@GO, H_2_O_2_ + AgNPs@Cs-TA, H_2_O_2_ + AgNPs@Cs-TA@GO, TMB + H_2_O_2_ + AgNPs@Cs-TA and TMB + H_2_O_2_ + AgNPs@Cs-TA@GO, all under identical assay conditions. The procedure for detecting H_2_O_2_ involved adding *m* mg of AgNPs@Cs-TA@GO composites to a cuvette with 200 μl of H_2_O_2_ at different concentrations and 2 ml of TMB (0.5 mM). The mixture was incubated for 5 min at ambient temperature, and then the absorbance was measured at 657 nm. The linear relationship of H_2_O_2_ was analysed in the concentration range of 0 to 36 ppm. The limit of detection (LOD) was calculated using the formula 3*σ*/*s*, where *s* is the slope of the linear curve and *σ* is the standard deviation.

### 2.9. Determination of H_2_O_2_ residue in aquaculture water

The presence of H_2_O_2_ residue in aquaculture water samples was identified through the standard addition method. Water samples were obtained from an aquaculture pond in Thanh Phu district, Ben Tre province, Vietnam. The detection process adhered to a pre-existing method [44] with slight adjustments as follows. The water sample was then filtered using a Whatman paper with a pore size of 0.45 μm. Recovery studies were conducted by adding specific concentrations of H_2_O_2_ to aquaculture water samples for testing. The concentration of added H_2_O_2_ fell within the linear range of absorbance and hydrogen peroxide. The experiment involved analysing aquaculture water samples treated with H_2_O_2_, TMB and the peroxidase mimic AgNPs@Cs-TA@GO to detect the presence of H_2_O_2_. The results of the H_2_O_2_ assay were also compared with the titration method involving KMnO_4_.

## 3. Results and discussion

### 3.1. Optimization and characterization of the designed composite

**Figure 1.**
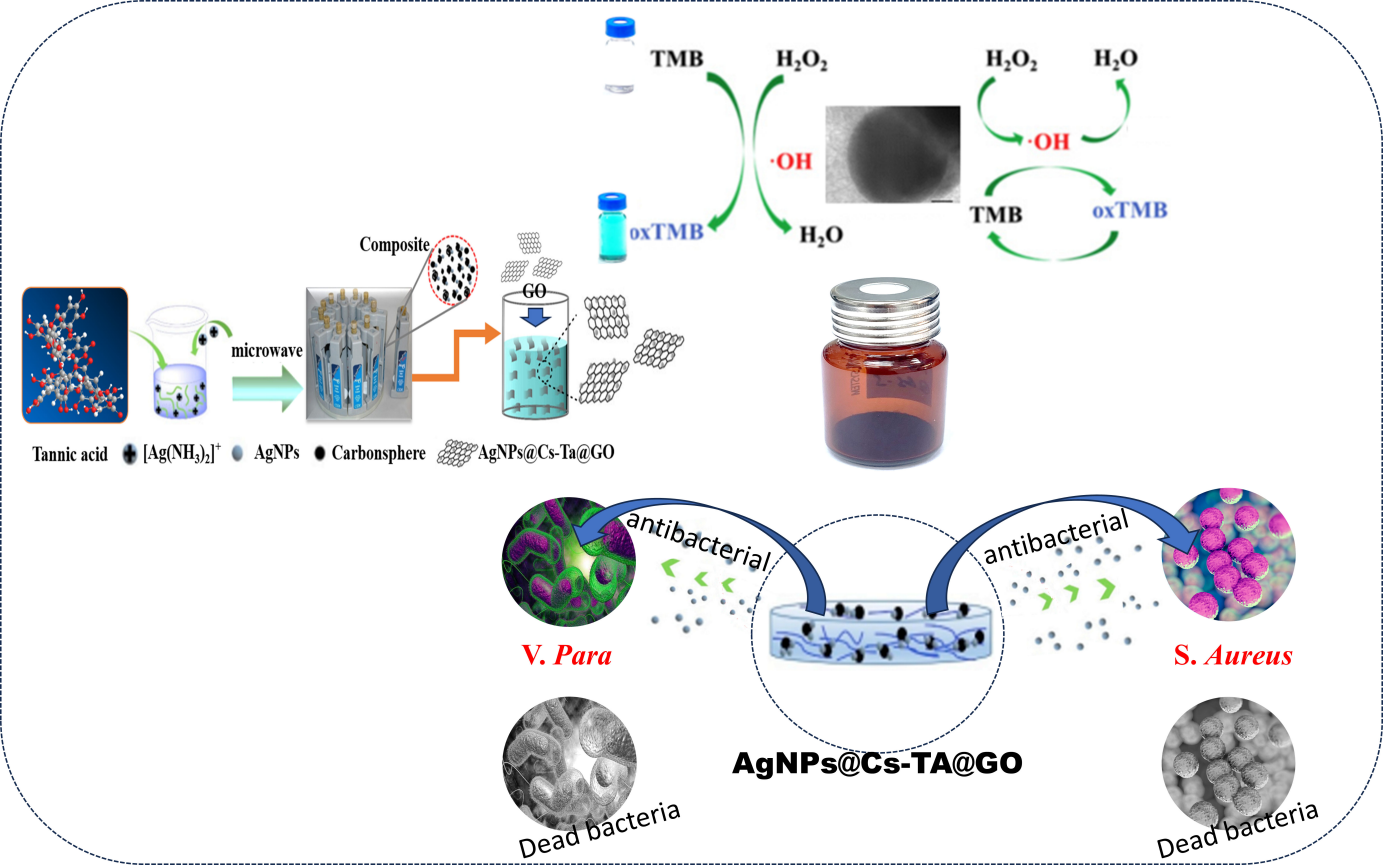
Schematic representation of the AgNPs@Cs-TA@GO synthesis and their application.

Three separate steps are involved in the development of AgNPs@Cs-TA@GO composite. In the first step, Cs are produced by using pure TA. Second, the silver ions are reduced, forming clusters that deposit into the Cs, achieving a more stable state owing to the inhibition of further combinations between AgNPs. Finally, the synthesized AgNPs@Cs-TA is anchored onto GO sheets to minimize the aggregation tendency of the AgNPs@CS-TA spheres and enhance the electron conductivity of the composite, thereby improving catalytic efficiency and antibacterial properties. Figure 1 illustrates the process of designing AgNPs integrated with Cs and then anchoring them onto GO sheets, as well as the application of the final product.

The presence of AgNPs and their stability play a crucial role in antibacterial and catalytic applications. Various parameters such as microwave power, irradiation time and the weight ratio (w/w) of silver nitrate to TA were adjusted to optimize the synthesis of AgNPs@Cs-TA. Similarly, ratios of GO to AgNPs@Cs-TA were altered to enhance the synthesis of AgNPs@Cs-TA@GO. XRD and zeta potential measurements were conducted to determine the optimal synthesis parameter (electronic supplementary material, figures S1 and S2). It has been found that the most appropriate synthesis condition was determined to be a microwave power of 50%, irradiation time of 2 min, a silver nitrate to TA ratio of 1 : 5 and a weight percentage of GO of 20%.

The optimal synthesis conditions were established based on XRD and zeta potential analyses. As shown in the electronic supplementary material, figure S1a, 50% microwave power produced well-defined AgNPs diffraction peaks and the highest colloidal stability (−74.5 mV), while higher powers, though also yielding crystalline AgNPs, led to reduced stability and unnecessary energy consumption. The electronic supplementary material, figure S1b demonstrates that a reaction time of 2 min is sufficient for AgNP crystallization, with no peaks observed at 0 min and no significant improvement beyond 2 min. Zeta potential data (−34.9 mV) further confirm the stability of the product at this duration.

As shown in the XRD patterns and zeta potential measurements in the electronic supplementary material, figure S2a, an AgNO_₃ _: TA w/w ratio of 1 : 5 resulted in the most distinct diffraction peaks corresponding to metallic silver, indicating efficient reduction and nanoparticle crystallization. This ratio also provided the highest colloidal stability (−39.9 mV), whereas both lower and higher ratios led to diminished crystallinity or reduced stability. Similarly, varying the GO weight content from 5 to 30% revealed that 20% GO offered the optimal balance between structural order and electrostatic stabilization, exhibiting sharp XRD peaks and the highest zeta potential (−42.5 mV; electronic supplementary material, figure S2b).

**Figure 2.**
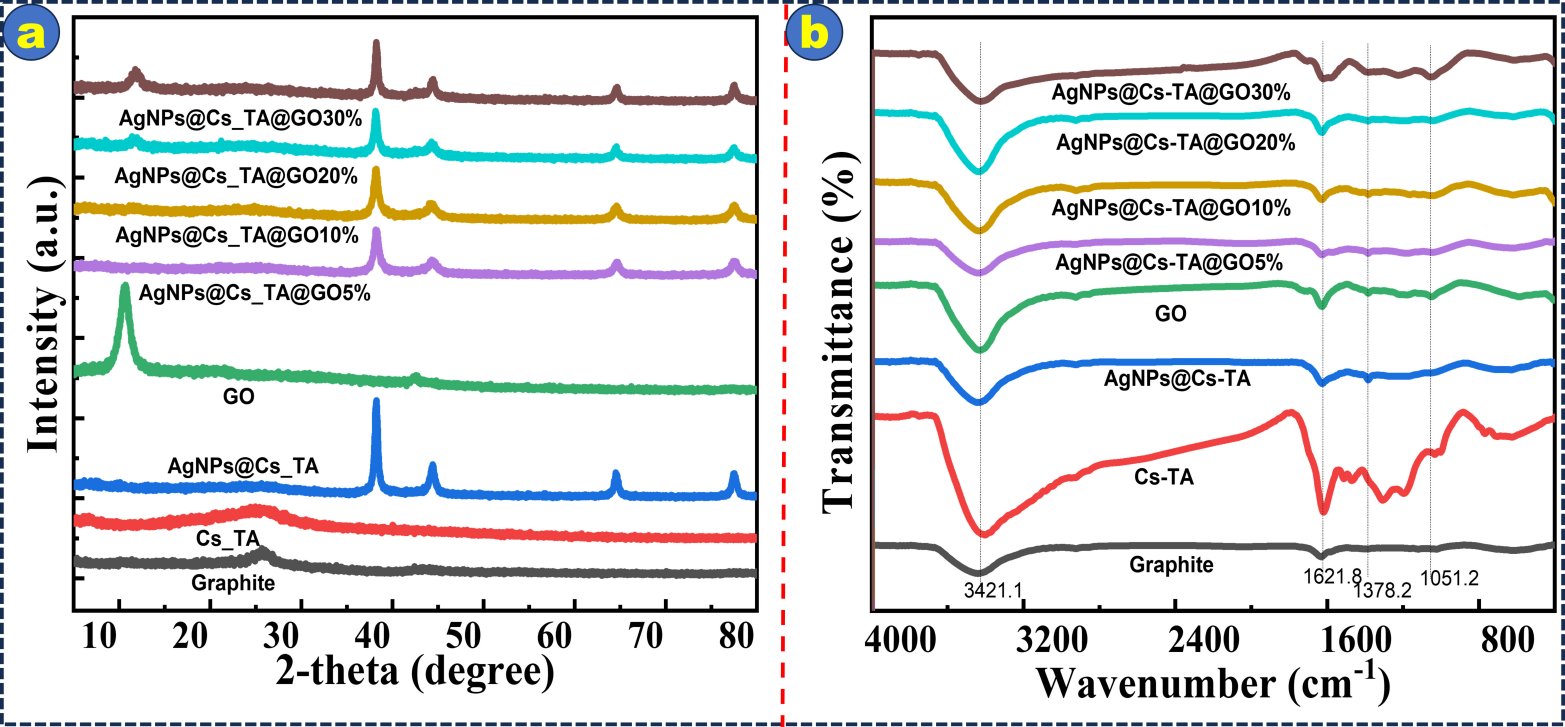
XRD patterns (a), and FTIR (b) spectra of graphite, tannic acid-based carbon spheres, GO, AgNPs@Cs-TA and AgNPs@Cs-TA@GO with GO at various ratios.

Figure 2 displays XRD patterns and FTIR spectra illustrating the structural features of graphite, Cs-TA, GO, AgNPs@Cs-TA and AgNPs@Cs-TA@GO with different GO ratios. The XRD patterns in figure 2a confirm the successful synthesis of each component. The sharp peaks at 2θ = 38.2, 44.3, 64.6 and 74.6° indicate the presence of AgNPs, which correspond to the crystallographic planes of cubic AgNPs. A prominent peak at 2θ = 10° confirms the presence of GO sheets. Cs have an amorphous shape and do not show distinct sharp peaks on the XRD pattern. Their distinctive peaks are hidden by the wide amorphous peak occurring between 20 and 30°. In AgNPs@Cs-TA@GO samples with a GO ratio exceeding 20%, AgNPs embedded in Cs and subsequently affixed to the surface of the GO sheets have been observed and identified. This observation is corroborated by the GO peak’s conspicuous visibility. At a GO percentage below 20%, however, the distinctive peak that is characteristic of GO sheets becomes undetectable. The observed phenomenon may be ascribed to the enhanced crystallinity of AgNPs relative to GO, whereby the intense presence of AgNPs might obscure that of GO.

Figure 2b displays the FTIR spectra showing the unique chemical compositions of graphite, Cs-TA, GO, AgNPs@Cs-TA and AgNPs@Cs-TA@GO at different GO ratios. The absorption peak at 3421.1 cm^−1^ represents the stretching vibrations of OH groups, which are notably present in all samples. The bands seen at 1621.8, 1378.2 and 1051.2 cm^−1^ indicate the existence of C=C, epoxy C–O groups and alkoxy C–O bonds, respectively. A noticeable reduction in the intensity of the modified GO peaks compared with pure GO indicates a decrease in GO functional groups when exposed to AgNPs@Cs-TA. The decrease in peak intensity suggests a modification in the chemical surroundings of the GO sheets, probably owing to interactions with the AgNPs@Cs-TA composite. The interaction between AgNPs@Cs-TA and the functional groups of GO supports the stability and enhances the antibacterial and catalytic efficiency of the composite material, especially after numerous cycles of reuse.

**Figure 3.**
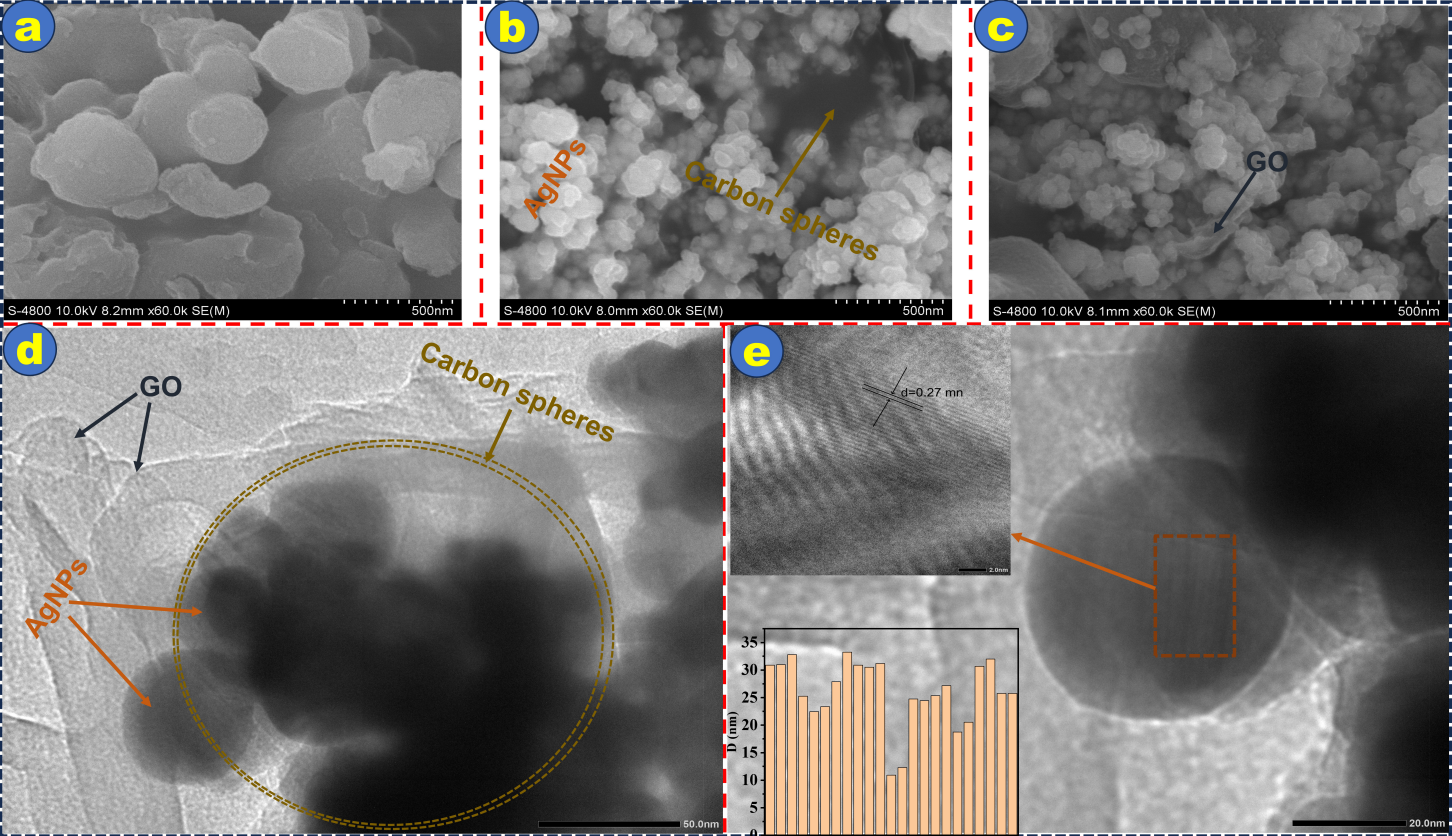
SEM images of (a) Cs-TA, (b) AgNPs@Cs-TA, (c) AgNPs@Cs-TA@GO, (d) TEM, and (e) HR-TEM images of AgNPs@Cs-TA@GO at 50 and 20 nm resolution, respectively. The inset in (e) presents the particle size distribution and an HR-TEM image of AgNPs@Cs-TA@GO at 2 nm resolution, highlighting the d-spacing corresponding to the (111) diffraction plane of AgNPs.

Figure 3 provides a detailed morphological and structural characterization of the as-synthesized composite. FE (Field Emission)-SEM images in figure 3a–c illustrate the stepwise formation of the composite structure. Figure 3a shows the spherical morphology of TA-derived Cs (Cs-TA) with relatively smooth surfaces. Upon the deposition of AgNPs onto the Cs (figure 3b), nanoparticles with diameters smaller than 50 nm become visible on the surface, indicating the successful loading of AgNPs. In figure 3c, after the incorporation of GO, the AgNPs@Cs-TA particles are observed to be well dispersed over thin GO sheets, forming the final AgNPs@Cs-TA@GO composite. This structural configuration suggests strong interactions and good compatibility among the three components.

Furthermore, structural details are revealed in the TEM and HR-TEM images. Figure 3d displays a TEM image of the AgNPs@Cs-TA@GO composite at 50 nm resolution, clearly confirming the presence of AgNPs both embedded within and distributed on the surface of the porous Cs. These Cs are anchored onto the GO sheets, providing visual evidence of the successful integration of all three components.

The high-resolution TEM image in figure 3e, captured at 20 nm resolution, shows distinct lattice fringes of AgNPs, indicating their crystalline structure. The lower left inset presents the AgNPs’ particle size distribution, with an average particle diameter of approximately 21 nm. The upper left inset highlights a lattice spacing of 0.235 nm, which corresponds to the (111) diffraction plane of face-centred cubic AgNPs. This nanoscale observation is consistent with the crystallite size of approximately 23 nm estimated from XRD data using the Debye–Scherrer equation (equation (2.1)).

**Figure 4.**
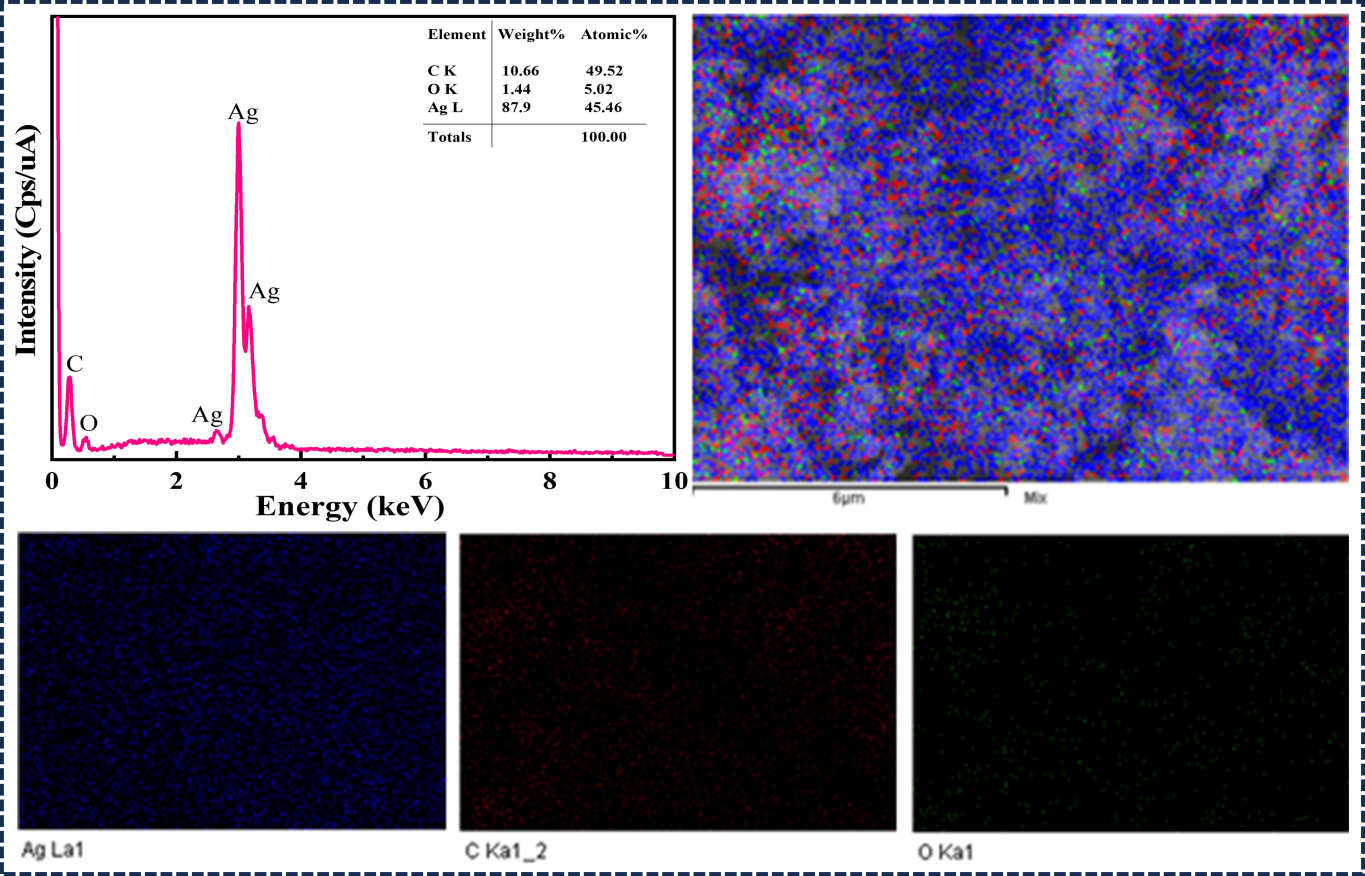
EDX spectrum and elements mapping images of AgNPs@Cs-TA@GO sample.

The chemical composition in AgNPs@Cs-TA@GO composite was verified by EDX analysis (figure 4). The analysis reveals that the weight percentage of carbon is 49.52%, silver is 45.46% and oxygen is 5.02%. The element mapping images show an even distribution of nanoparticles across the GO sheets (figure 4). This discovery provides more proof of nanoparticles adhering to both the carbon sphere and the GO network. Metal nanoparticles can interact with GO sheets by physisorption, electrostatic binding or charge-transfer interactions. The uniform dispersion of silver elements indicates that the material will possess effective antibacterial and catalytic characteristics.

**Figure 5.**
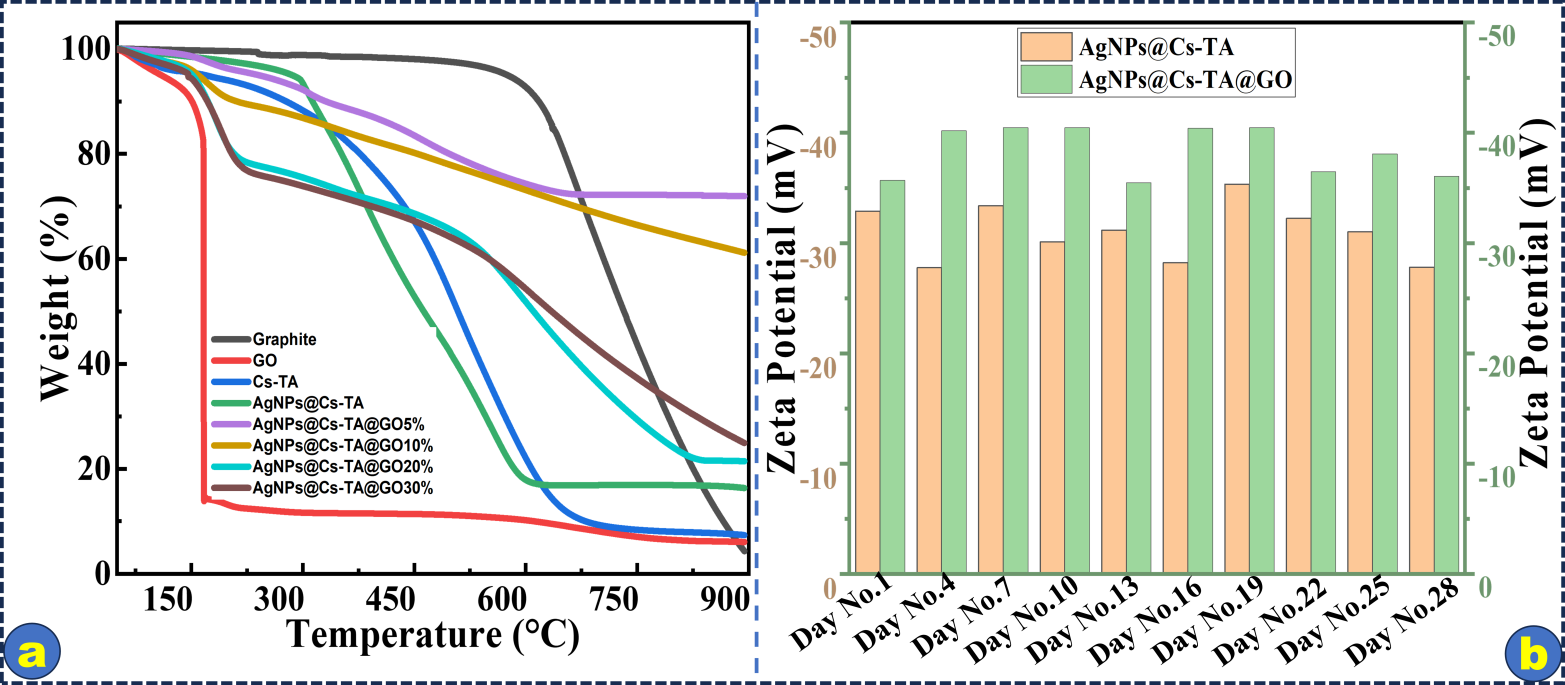
TGA curves of AgNPs@Cs-TA@GO composite and their components (a), and zeta potentials of AgNPs@Cs-TA and AgNPs@Cs-TA@GO over time (b).

Figure 5a shows TGA curves of Cs-TA, AgNPs@Cs-TA and AgNPs@Cs-TA@GO nanocomposites with different ratios of GO under a nitrogen atmosphere. A comparison analysis of thermal characteristics acquired from TGA is undertaken to accurately identify differences in the thermal degradation behaviour among the samples. The first weight loss in Cs-TA is owing to the elimination of adsorbed water by hydrophilic groups. Chain scission and ring-opening reactions are the primary degradation processes that occur at temperatures under 600°C. Approximately 10% of carbon residue is detected after the thermal degradation of Cs-TA at 600°C in a nitrogen environment. GO derivatives display two distinct stages of weight reduction. AgNPs@Cs-TA has a complex degradation profile with at least three phases during degradation owing to the presence of the inorganic filler of AgNPs. AgNPs@Cs-TA@GO5% and AgNPs@Cs-TA@GO10% exhibit two deterioration stages, while AgNPs@Cs-TA@GO20% and AgNPs@Cs-TA@GO30% show three degradation stages. Complete weight loss occurs at the last stage owing to the breakdown of intermediates generated in earlier phases. At 600°C, just 2% of carbon residue remains, which is linked to the burning of polysaccharide components in the presence of nitrogen. AgNPs@Cs-TA@GO20% and AgNPs@Cs-TA@GO30% samples show similar degradation patterns, unlike AgNPs@Cs-TA@GO5% and AgNPs@Cs-TA@GO10%.

The DLS technique was used to assess the surface zeta potential and dispersity of the synthesized AgNPs in an aqueous colloidal solution. In figure 5b, the average negative zeta potential values of −32.27 mV for AgNPs@Cs-Ta and −35.69 mV for AgNPs@Cs-TA@GO were observed. The more negative zeta potential caused by GO contributes to the total repulsion among nanoparticles, effectively preventing agglomeration. The zeta potential values remained almost unchanged after 30 days, confirming the enhanced stability of the AgNPs@Cs-TA@GO sample.

### 3.2. Antibacterial activity

**Figure 6.**
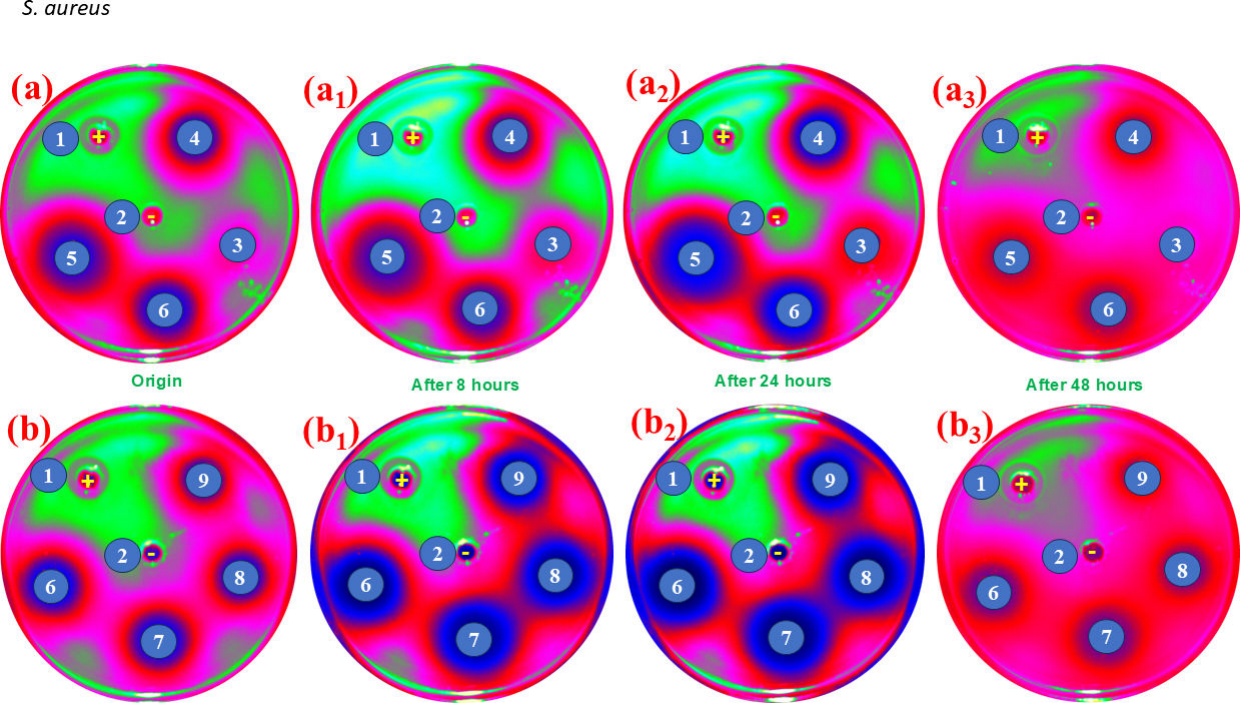
Time-dependent antibacterial efficacy against *S. aureus:* (a, a_1_−a_3_) using the composite and its constituents: (1) ClO_2_ (positive control), (2) H_2_O (negative control), (3) AgNO_3_@Cs-TA 1%, (4) Cs-TA 20%, (5) AgNPs@Cs-TA 50 ppm and (6) AgNPs@Cs-TA@GO 50 ppm; (b, b_1_−b_3_) inhibition zones of AgNPs@Cs-TA@GO at different concentrations: (6) 25 ppm, (7) 12.5 ppm, (8) 6.25 ppm and (9) 4.16 ppm. The dark blue region at the centre represents the diffusion zone of the composite, the pink red region indicates strong antibacterial inhibition and the green region corresponds to areas of bacterial growth.

**Figure 7.**
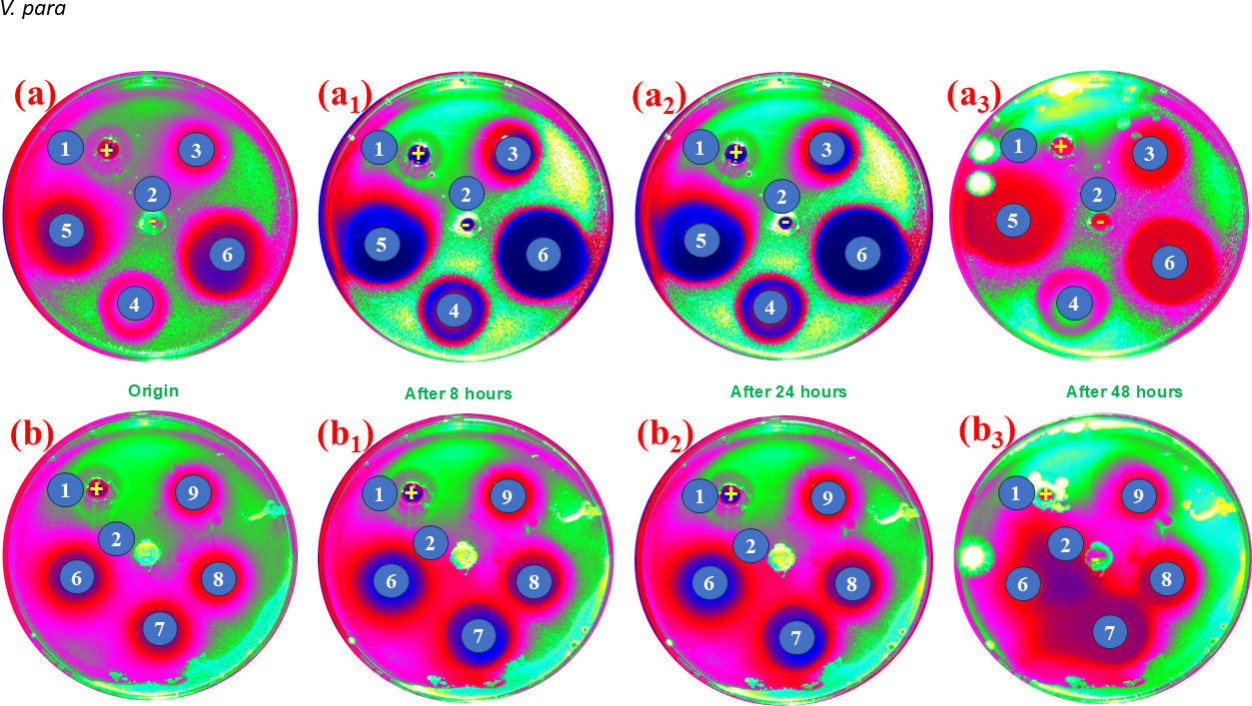
Time-dependent antibacterial efficacy against *V. para:* (a, a_1_−a_3_) using the composite and its constituents: (1) ClO_2_ (positive control), (2) H_2_O (negative control), (3) AgNO_3_@Cs-TA 1%, (4) Cs-TA 20%, (5) AgNPs@Cs-TA 50 ppm and (6) AgNPs@Cs-TA@GO 50 ppm; (b, b_1_−b_3_) inhibition zones of AgNPs@Cs-TA@GO at different concentrations: (6) 25 ppm, (7) 12.5 ppm, (8) 6.25 ppm, (9) 4.16 ppm. The dark blue region at the centre represents the diffusion zone of the composite, the pink red region indicates strong antibacterial inhibition and the green region corresponds to areas of bacterial growth.

The comparative antibacterial effectiveness of Cs-TA, AgNO_3_@Cs-TA, AgNPs@Cs-TA and AgNPs@Cs-TA@GO composites was thoroughly assessed by analysing their ability to inhibit bacterial growth on agar medium using the agar well diffusion method. The quantitative bactericidal capabilities were evaluated against *V. para* and *S. aureus* at a bacterial concentration of 10^8^ CFU ml^−1^. The agar well diffusion test findings are presented in figures 6 and 7 and the electronic supplementary material, table S1, figures S3 and S4. The results show that the composite and its components effectively inhibited the growth of both bacterial strains (figures 6 and 7). The AgNPs@Cs-TA@GO nanocomposite showed the most outstanding growth inhibition properties. The mean inhibition zones generated by AgNPs@Cs-TA@GO (50 ppm) against *S. aureus* and *V. para* were 49.3 ± 0.17 and 46.1 ± 0.11 mm, respectively. The diameters induced by AgNPs@Cs-TA (50 ppm) were 28.6 ± 0.16 and 27.4 ± 0.13 mm, by AgNO_3_@Cs-TA (1%) were 20.3 ± 0.21 and 22.3 ± 0.16 mm and by Cs-TA (20%) were 17.2 ± 0.17 and 18.4 ± 0.14 mm. The AgNPs@Cs-TA@GO composite showed stronger antibacterial effects against *S. aureus* than *V. para* bacteria. The composite successfully suppressed the growth of both strains for up to 48 h. The TA compound used in the manufacture of AgNP functions facilitates robust attachment of each AgNP to Cs and GO surfaces and preserves their monodispersity, leading to prolonged and consistent antibacterial effects. Compared to other materials, the composite also demonstrates its outstanding antibacterial properties (table 1).

**Table 1.** Comparative antibacterial activity of AgNPs from different resources. The bold text highlights the materials synthesized in our current study.

bacterial strains	material	AgNPs size (nm)	concentration of AgNPs (ppm)	zone of inhibition (mm)	ref.
*S. aureus*	AgNPs using *Psuedomonas koreensis* THGLS1.4	10–40	500	16.0 ± 0.2	[45]
	silver–graphene oxide using walnut green husk extract	41 ± 20	25	18	[46]
	chitosan/gelatin/AgNP composites	10–30	1000	19	[47]
	silver-doped TiO_2_ nanoparticles using aqueous extract of *Acacia nilotica*	9	500	20	[48]
	AgNPs using *Anoectochilus elatus* extract	22.48	100	20 ± 1.6	[49]
	AgNPs using *Spirulina* extract	10–40	10	16.0 ± 0.2	[50]
	**AgNPs@Cs-TA@GO**	**21 ± 12**	**50**	**49.3 ± 0.17**	**this research**
*V. para*	AgNPs using *Psuedomonas koreensis* THGLS1.4	10–40	500	16.5 ± 0.5	[45]
	AgNPs using coconut inflorescence sap	10–30	6.8	20 ± 1.1	[51]
	AgNPs producing bacteria	8–30	12.5	21.4 ± 1.5	[52]
	AgNPs using *Anoectochilus elatus* extract	22.48	100	18 ± 1.44	[49]
	AgNPs using *Spirulina* extract	10–40	10	16.5 ± 0.5	[50]
	**AgNPs@Cs-TA@GO**	**21 ± 12**	**50**	**46.1 ± 0.11**	**this research**

The antibacterial activity of the AgNPs@Cs-TA@GO composite arises from the synergistic effects of AgNPs, Cs and GO. AgNPs disrupt bacterial membranes, penetrate cells to damage vital components, generate reactive oxygen species (ROS) and interfere with signal transduction [53]. Cs enhance the dispersion and stability of AgNPs, promoting effective contact with bacteria and contributing to ROS generation [54]. GO further improves antibacterial efficacy by physically damaging bacterial membranes, adsorbing cells to block nutrient exchange and amplifying oxidative stress [55,56].

To evaluate the biocompatibility and biosafety of the AgNPs@Cs-TA@GO composite for antimicrobial applications, the composite’s cytotoxicity was assessed on human dermal fibroblast cells (ATCC, USA) using the sulforhodamine B assay [57]. The results, summarized in the electronic supplementary material, table S2, show that at 5.0 ppm, the composite exhibited negative cytotoxicity (−5.64%), indicating no toxic effect and potentially promoting cell growth. At 2.5 ppm, the cytotoxicity increased to 11.16%, which remained significantly lower than the positive control (camptothecin, 44.65%) and comparable to the negative control (distilled water, 4.42%). Note that the lowest effective antibacterial concentration was 4.16 ppm, and cytotoxicity remains low at this range; the composite can be considered safe for use in aquaculture environments, such as shrimp pond water treatment, where direct exposure to mammalian cells is minimal.

### 3.3. Peroxidase-like activity and assay of H_2_O_2_

**Figure 8.**
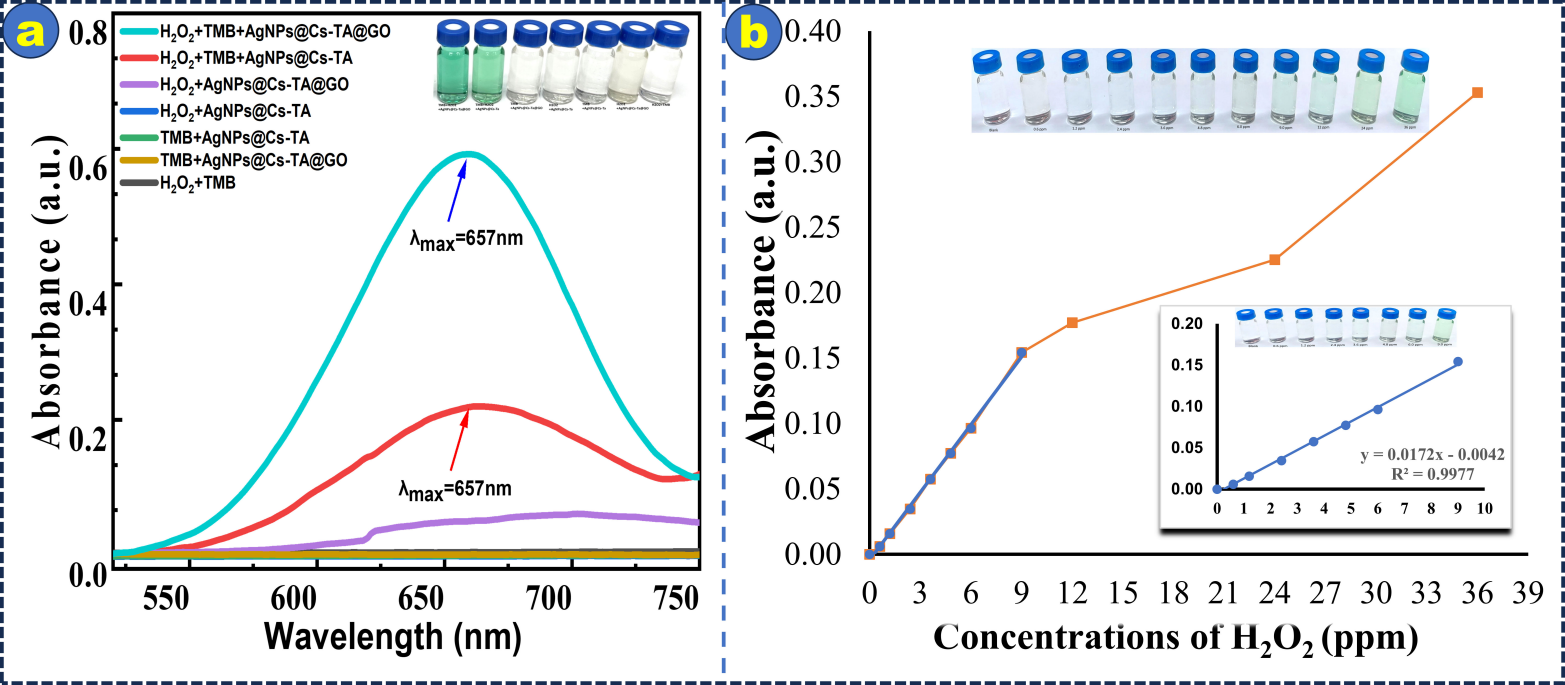
(a) UV–Vis absorption spectra comparing the peroxidase-like activity of the AgNPs@Cs-TA@GO nanocomposite with its components. The inset in (a) shows the corresponding colour changes of each system and (b) shows the calibration curve of absorbance at 657 nm versus H_2_O_2_ concentration. The inset in (b) shows a linear region in the concentration range of 0–9 ppm. The inset also shows the visible colour changes of the solution with increasing H_2_O_2_ concentrations.

The peroxidase-like catalytic properties of the AgNPs@Cs-TA@GO composite were evaluated using TMB as a colorimetric substrate in the oxidation reaction with or without H_2_O_2_ (figure 8). The UV–Vis absorbance between 500 and 800 nm was observed. Figure 8a shows individual reaction systems with TMB + H_2_O_2_, TMB + AgNPs@Cs-TA, TMB + AgNPs@Cs-TA@GO, H_2_O_2_ + AgNPs@Cs-TA and H_2_O_2_ + AgNPs@Cs-TA@GO, all displaying low and insignificant absorbance. The H_2_O_2_ + TMB + AgNPs@Cs-TA and H_2_O_2_ + TMB + AgNPs@Cs-TA@GO systems showed a more pronounced absorption band at 657 nm, demonstrating effective catalysis of TMB oxidation and the generation of oxidized TMB as a blue-coloured product by H_2_O_2_. The H_2_O_2_ + TMB + AgNPs@Cs-TA@GO system turned dark blue in 40 s, whereas the other systems stayed colourless or had weak colour intensity. The observation indicates the superior catalytic activity compared to its components. The enhanced catalytic activity of the AgNPs@Cs-TA@GO nanocomposite in the presence of H_₂_O_₂_ can be attributed to its well-engineered hierarchical architecture. Specifically, AgNPs are embedded within porous Cs, providing primary physical confinement that limits their mobility and aggregation. These Cs are subsequently anchored onto GO sheets, which contribute a secondary level of stabilization. The GO matrix, with its high surface area and abundant oxygen-containing functional groups [20], enhances the dispersion of AgNP-loaded Cs by preventing their agglomeration. Furthermore, the synergistic integration of AgNPs, Cs and GO sheets creates more efficient electron transport pathways within the composite [20], which is critical for enhancing its peroxidase-like catalytic activity.

The proposed catalytic mechanism proceeds through two key steps. First, AgNPs from AgNPs@Cs-TA@GO are oxidized by hydrogen peroxide at pH 7, leading to the formation of silver ions and hydroxyl ions [58], as shown in equation (2.2):


(2.2) $$H_{2}O_{2}+2Ag^{0}\to 2OH^{-}+2Ag^{+}.$$

In the second step, the generated Ag^+^ ions subsequently oxidize the reduced form of TMB (TMB_Red_) to its oxidized form (TMB_Ox_). This reaction is driven by the fact that the standard redox potential of Ag^+^/Ag⁰ (0.80 V) is significantly higher than that of TMB_Red_/TMB_Ox_ (0.22 V) [59], as described in equation (2.3):


(2.3) $$Ag^{+}+TMB_{Red}\to Ag^{0}+TMB_{Ox}.$$

Notably, the AgNPs@Cs-TA@GO nanocomposite demonstrated superior catalytic performance compared with AgNPs@Cs-TA, which can be attributed to the potential role of GO as an electron mediator [18] between the electron donor (TMB_Red_) and the electron acceptor (Ag^+^).

The detection limit for H_2_O_2_ was found to be 0.2 ppm with a signal-to-noise ratio of 3, and it has a broad linear range from 0.6 to 9 ppm (figure 8b). Table 2 presents a comparison of various materials used for hydrogen peroxide detection. Among them, the developed AgNPs@Cs-TA@GO composite demonstrates a notably low LOD of 5.9 μM, indicating a higher sensitivity compared with other colorimetric methods. For instance, Fe/CuSn(OH)₆, FePt-Au HNPs and Au@Pt NRs exhibit higher LODs of 9.49, 12.3 and 45 μM, respectively. While some electrochemical methods, such as rGO/Ag NPs, offer broader linear ranges, their LODs tend to be higher (e.g. 31.3 μM), which may limit their sensitivity at low concentrations. By contrast, the AgNPs@Cs-TA@GO composite provides both a suitable linear range (17.7–265.5 μM) and superior sensitivity, making it a promising candidate for practical and accurate hydrogen peroxide sensing.

**Table 2.** Comparison of reported sensors for hydrogen peroxide detection. The bold text highlights the materials synthesized in our current study.

probe	method	linearity range	LOD	ref.
VO_2_ nanofibres	colorimetric	0.025–10 mM	0.018 mM	[60]
VO_2_ nanosheets	colorimetric	0.49–62.5 mM	0.27 mM	[60]
VO_2_ nanorods	colorimetric	0.49–15.6 mM	0.41 mM	[60]
Fe/CuSn(OH)_6_	colorimetric	30–1000 μM	9.49 µM	[44]
Au @ Pt NRs	colorimetric	45–1000 μM	45 µM	[61]
FePt-Au HNPs	colorimetric	20–700 μM	12.3 µM	[62]
rGO/Ag NPs	electrochemical	100–100000	31.3 µM	[63]
Pt-TiO_2_/RGO	electrochemical	0–20 mM	—	[64]
**AgNPs@Cs-TA@GO**	**colorimetric**	**17.7–265.5 µM** (**0.6–9 ppm**)	**5.9 μM (0.2 ppm**)	**this research**

### 3.4. Optimization of hydrogen peroxide detection

To determine the optimal catalytic conditions, the effects of pH (2–13), reaction time (0–60 min) and catalyst dosage (0.1–1.0 mg) on the peroxidase-like activity of AgNPs@Cs-TA and AgNPs@Cs-TA@GO were systematically investigated. Catalytic performance was assessed by monitoring the absorbance at 657 nm, which corresponds to the oxidation of TMB in the presence of H_₂_O_₂_. The optimal parameters for both systems were selected based on the highest absorbance values, indicating the most efficient generation of oxidized TMB. Among the tested conditions, the highest catalytic activity for AgNPs@Cs-TA@GO was observed at pH 7.0, a catalyst dosage of 0.2 mg and a reaction time of 5 min (electronic supplementary material, figure S5). By contrast, the AgNPs@Cs-TA system required a longer reaction time (25–60 min) to achieve a comparable absorbance.

### 3.5. Analysis of H_2_O_2_ residue in a real aquaculture water sample

The AgNPs@Cs-TA@GO colorimetric sensor was used to detect residual H_2_O_2_ in aquaculture water to confirm the reliability of the sensor in real-world scenarios. The test data are displayed in Table SI.3. The recoveries varied from 96.3 to 103.2% with a relative standard deviation ranging from 0.78 to 2.14%. Four samples of aquaculture water were found to contain H_2_O_2_, with concentrations ranging from undetectable to 1064.3 ppm. The results were in agreement with those obtained through titration with KMnO_4_. The results show that our method is highly reliable and has remarkable consistency for detecting H_2_O_2_ in aquaculture water.

## 4. Conclusions

In this study, the environmentally friendly green decoration of a nanocomposite based on AgNPs, Cs and GO was successfully designed using a combination of microwave-assisted technique and ultrasonic technology. The nanocomposite, with a core based on AgNPs averaging 21 ± 12 nm in diameter, exhibited superior peroxidase-like catalytic activity for TMB in the presence of H_2_O_2_. This susceptible H_2_O_2_ colorimetric detection method allowed for rapid identification of H_2_O_2_ within a wide range from 0.6 to 9.0 ppm, and a low detection limit of 0.2 ppm. The effectiveness of this method was confirmed by qualitatively detecting residual H_2_O_2_ in aquaculture water samples. Additionally, antibacterial tests of the composite also demonstrated higher inhibitory effects against *V. para* and *S. aureus* with antibacterial zones measuring 46.1 ± 0.11 mm and 49.3 ± 0.17 mm. The successful production of this environmentally friendly nanocomposite provides bright opportunities for future uses in catalysis, antibacterial treatments and sensitive hydrogen peroxide detection.

## Data Availability

The data supporting the findings of this study are available through Dryad [65]. Supplementary material is available online [66].
